# Boosting robot-assisted rehabilitation of stroke hemiparesis by individualized selection of upper limb movements – a pilot study

**DOI:** 10.1186/s12984-019-0513-0

**Published:** 2019-03-20

**Authors:** Orna Rosenthal, Alan M. Wing, Jeremy L. Wyatt, David Punt, Briony Brownless, Chit Ko-Ko, R. Christopher Miall

**Affiliations:** 1 School of Psychology, University of Birmingham, B15 2TT, Birmingham, UK; 2 School of Computer Science, University of Birmingham, B15 2TT, Birmingham, UK; 3 School of Sport, Exercise and Rehabilitation Sciences, University of Birmingham, B15 2TT, Birmingham, UK; 4 West Midlands Rehabilitation Centre, Birmingham, B29 6JA UK

**Keywords:** Stroke, Rehabilitation, Robot-assisted therapy, Upper limb movements, Reaching task

## Abstract

**Background:**

Intensive robot-assisted training of the upper limb after stroke can reduce motor impairment, even at the chronic stage. However, the effectiveness of practice for recovery depends on the selection of the practised movements. We hypothesized that rehabilitation can be optimized by selecting the movements to be practiced based on the trainee’s performance profile.

**Methods:**

We present a novel principle (‘steepest gradients’) for performance-based selection of movements. The principle is based on mapping motor performance across a workspace and then selecting movements located at regions of the steepest transition between better and worse performance.

To assess the benefit of this principle we compared the effect of 15 sessions of robot-assisted reaching training on upper-limb motor impairment, between two groups of people who have moderate-to-severe chronic upper-limb hemiparesis due to stroke. The test group (*N* = 7) received steepest gradients-based training, iteratively selected according to the steepest gradients principle with weekly remapping, whereas the control group (*N* = 9) received a standard “centre-out” reaching training. Training intensity was identical.

**Results:**

Both groups showed improvement in Fugl-Meyer upper-extremity scores (the primary outcome measure). Moreover, the test group showed significantly greater improvement (twofold) compared to control. The score remained elevated, on average, for at least 4 weeks although the additional benefit of the steepest-gradients -based training diminished relative to control.

**Conclusions:**

This study provides a proof of concept for the superior benefit of performance-based selection of practiced movements in reducing upper-limb motor impairment due to stroke. This added benefit was most evident in the short term, suggesting that performance-based steepest-gradients training may be effective in increasing the rate of initial phase of practice-based recovery; we discuss how long-term retention may also be improved.

**Trial registration:**

ISRCTN, ISRCTN65226825, registered 12 June 2018 - Retrospectively registered,

**Electronic supplementary material:**

The online version of this article (10.1186/s12984-019-0513-0) contains supplementary material, which is available to authorized users.

## Background

Upper-limb (UL) motor impairment is a common outcome of stroke that can severely hamper basic daily living activities [1–3]. Training-based therapy can promote recovery with the outcome depending on the intensity and duration of the intervention [4–6]. Robot-assisted training allows intense practice without increasing the individual’s dependence on a therapist and can improve clinical scores of UL motor capacity [7–9]. However, the effects are usually small and provide limited improvement in motor function, especially in more severe hemiparesis [6, 7, 10–12]. Identifying training methods that can boost outcome is thus vital. Considering the extent of effort and sophistication invested in robot-assisted technology (e.g. [13, 14]) perhaps it is time to focus on how to optimise its utility (in terms of training principles). Recent attempts have focussed on creating training scenarios which are more engaging or which simulate daily living activities. However, the evidence for the added benefit of this approach is mixed [15]. Another approach is to individualize the difficulty of the practised task (e.g. [16, 17]). This is based on the idea that motor improvement depends on the ability to ‘make sense’ of information related to performance [18], and postulates that matching the challenge (difficulty) level of the training task to the current ability of the trainee would optimise motor learning [19]. Individualizing task difficulty is commonly achieved by adjusting the parameters controlling task demands (e.g. movement speed or distance; or amount of assistance) across a pre-selected standard set of movements, to match the ability of the individual. Yet, so far there is little evidence for the added benefit of this approach for UL motor recovery. Hence, individually adjusting the task difficulty level might –by itself - not suffice for boosting UL rehabilitation outcome.

**Fig. 1:**
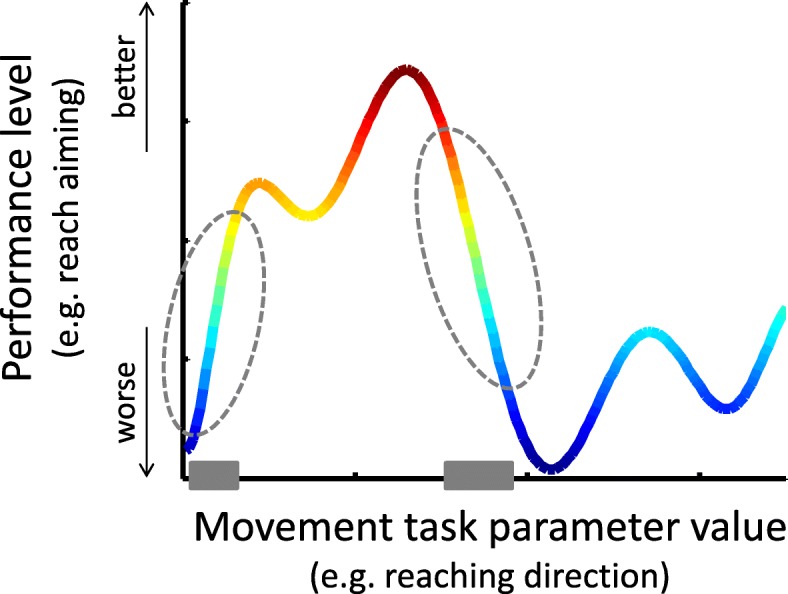
Illustrative sketch of the principle of selection of trained movements, based on the steepest gradients in a hypothetical motor performance profile (e.g. reaching aiming; vertical axis) measured across some particular task parameter (e.g. movement direction; horizontal axis); for simplicity, we show here a single dimension. The selected movements (grey horizontal bars) correspond to the regions with the steepest performance gradients, indicated by dashed ellipses. This movement selection principle can be applied where movement tasks can be defined by one or more continuous parameters, i.e. in a 1D, 2D, or higher dimensional map as long as the derivative of performance can be calculated. In this study we applied this principle on two measures of reaching performance (ability to move and ability to aim) each measured across two dimensions of the task (target location and movement direction)

We hypothesised instead that appropriate selection of the practiced movements – in terms of the muscle coordination patterns - is a key for improving motor recovery. UL hemiparesis can affect various aspects of control. Thus, different motor impairments may benefit from different training movements. For example, training with movements involving mainly patterns of intact muscle coordination is unlikely to contribute much to improve other impaired movement patterns, regardless of the task difficulty level. Similarly, training that focuses only on movements that involve severely impaired muscle control may contribute little, even if the task can be performed by compensatory movements. Hence, to be optimally effective, individualized training may need to be expressed, not only by individually adjusting the level of difficulty of the task, but also in selecting tasks which are relevant for recovery. Little has been done to explore this possibility (for some attempts see [20, 21]). Here we present a novel method for performance-based selection of the set of movement tasks for robot-assisted training. The method depends on the availability of a motor performance “map” that profiles performance across a workspace. Movements are selected within intermediate levels of performance, based on the variation of performance across the map. Specifically, we predicted that optimal reduction of UL hemiparesis would be achieved by training with movements located at points on the map of steep transition (steep gradient) from high to low performance (Fig. 1), thus promoting the cascade of generalisation of motor improvement. Improved performance of movements at these steep gradient locations on the performance map would steer improvement in neighbouring, but more impaired regions, and encourage recovery. Here, we present evidence supporting this hypothesis.

To apply our method we first developed a novel principle of mapping of robot-assisted reaching performance across two dimensions of target location and movement direction [22], informing us about postural and movement-related aspects of motor control, respectively—key factors in the planning and execution of reaching movements [23–25]. The performance maps then served to select movement sets for training, based on our “steepest gradients” principle. To test our hypothesis–namely, training based on that principle would lead to superior recovery–we compared the outcome of 15 sessions of robot-assisted training between two groups of people who have severe-to-moderate chronic UL hemiparesis due to stroke, differing only in the selection of trained movement. In one group the selection was based on the steepest performance gradients principle (updated weekly) whereas the other group was trained with a fixed set of centre-out reaching movements regardless of participant’s performance profile, as commonly used in robot-assisted UL therapy [26].

## Methods

### Participants

We studied 16 adult individuals with chronic moderate-to-severe UL hemiparesis (Table 1).

**Table 1:** Participant details

participant	age	sex	hand		years since last stroke	Brain Lesion locations	FMA-UE (baseline)
			dominant	affected			
C1	65	F	R	R	2.5	L BG, L thal	11
C2	68	M	R	R	7	L front-par, L temp, L insula, L BG	40.5
C3	59	M	R	L	4	R par, R front	9.5
C4	43	F	R	L	2	R front, R par-temp, R hipp atrophy	47
C5	48	F	R	R	4	L hemisphere^a^	27
C6	20	F	R	R	2.5	L hemisphere (very large lesion)^a^	16
C7	75	M	R	L	10.5	R thal, R BG	20
C8	65	M	R	R	1	bilateral BG, bilateral thal, EnV	17
C9	58	M	R	R	3.5	L front-par (large lesion), L occ, L thal, L BG, EnV (especially L)	18
T1	65	F	R	R	3	L BG, L hipp atrophy	26.5
T2	60	M	L	R	3	L front-par, L insula, L temp	38
T3	51	M	R	L	3	R hemisphere lesion^a^	7.5
T4	57	M	R	R	9	L front-temp, L thal, L BG	14
T5	58	M	R	R	1	L thal, L CC (anterior), WM atrophy	12
T6	60	M	L	L	2.5	R BG, R thal, R EnV, R temp	10.5
T7	79	F	R	R	4.5	L BG, L thal, L EnV, L insula atrophy	20.5

C1–9: control; T1–7: test. Here FMA-UE score is the average across two baseline assessments. Anatomical abbreviations: *BG* basal ganglia, *CC* corpus callosum, *EnV* enlarged ventricles, *front* frontal cortex, *hipp* hippocampus, *occ* occipital cortex, *par* parietal cortex, *temp* temporal cortex, *thal* thalamus, *WM* white matter

^a^Based on family doctor’s notes

The study was conducted at the University of Birmingham (UK; the School of Psychology and Birmingham University Imaging Centre). Candidate participants were recruited via advertisements and visits to stroke clubs around Birmingham, as well as by contacts with potential recruits involved in other unrelated research at the University of Birmingham, who had provided written consent to be contacted for other projects.

Screening and initial (baseline) clinical assessments were conducted in two initial sessions by an experienced therapist. Inclusion criteria included: 1) aged 18+ years, 2) cortical or capsular stroke > 6 months before participation and no evidence for another stroke in the last 6 month, 3) Fugl-Meyer assessment (FMA-UE) [27] score within 5 and 50 points, with no more than 5-point difference on repeat testing at two weeks interval, 4) preserved vision across the visual field, allowing detection of all the stimuli displayed during the robot-assisted motor tasks, 5) ability to maintain balance when seated, 6) preserved basic cognitive function including understanding instruction as assessed by Mini Mental State Examination, 7) availability during the full period of the study.

Exclusion criteria included 1) prolonged pain in the affected upper limb or during movement (assessed using the 10-point Likert Pain Scale) or injury in the hemiparetic hand/arm, 2) severe spasticity involving elbow/shoulder movements ≥3 in Modified Ashworth Scale for any tested elbow/shoulder posture, 3) undergoing active rehabilitation (e.g. physiotherapy, occupational therapy etc.) during the study period, 4) cerebellar lesion assessed by MRI or by clinical report as provided by the participant.

Seventeen out of the 36 screened candidate participants were found eligible to take part in the study. One participant withdrew from the study before completing all the sessions and hence his data were excluded from this report.

All participants received detailed information about the study, which was approved by University of Birmingham local ethics committee, and gave informed consent (signed by themselves or by a trusted representative).

Three participants were not MRI-eligible and instead provided a copy of their clinical report about the lesioned hemisphere.

### Design, materials, and procedures

The study had a parallel design. Following screening and initial clinical assessment (CA) 17 participants were initially allocated to a control (*N* = 9) or test (*N* = 8) group, through a stratification algorithm aiming to balance age, UL impairment level (FMA-UE score), and handedness relative to the affected limb. One participant in the test group discontinued his participation, leaving the final test group *N* = 7.

Specifically, allocation to either test or control study group was done using stratification (a dynamic minimization protocol; conducted and updated using an Excel routine), balancing impairment (2 levels: FMA-UE score more or less than 25), age (two levels: younger or older than 60) and handedness with respect to the affected limb (2 levels: dominant or non-dominant) between the groups. Each participant, when recruited, was allocated to the group which had more stratification factors containing fewer participants with the same stratification as the incoming participant. If all three factors were balanced between the groups, allocation was based on a pre-set alternating list.

**Fig. 2:**
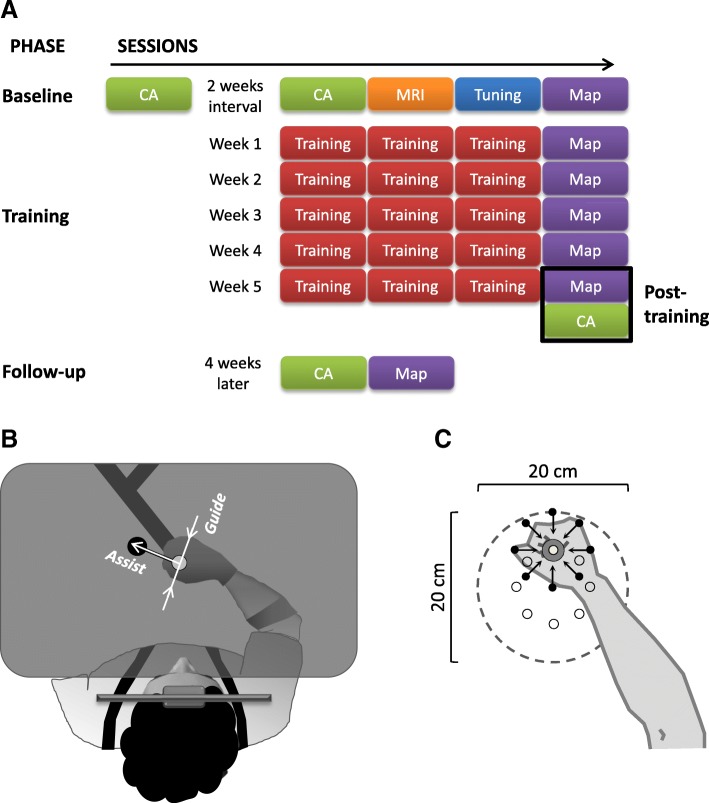
Experimental design. **a.** The sessions in each of the 3 participation phases are shown, with different colours indicating different session type. CA: clinical assessment; Map: *mapping* session. The first CA also served for screening. **b.** Schematic description of the experimental setting (top view; adapted from [32]). The participant held the robot handle, with grip ensured by a glove (Active Hands Co Ltd) and arm supported against gravity (SaeboMass, Saebo Inc.; not shown), which—at the beginning of each trial – was gently moved by the robot to a start position (white on-screen disc). Next, a target appeared on the horizontal display (blue on-screen disc; here shown black) and the participant tried to reach the target within the allotted time as accurately as possible, with the robot providing assisting and guiding forces as needed at each moment. Hand position was indicated on-screen by a red disc (not shown here). The horizontal display occluded the hand and the manipulandum from vision. Participants wore a harness to restrict trunk movement, keeping their forehead on a padded headrest attached to the workstation frame. The assistive force (*Assist*) promoted slower-than-allowed movements and also impeded very fast rebound-like movements characterising high elbow flexor muscle tone. The guiding force (*Guide*) impeded lateral deviation from a straight path towards the target. An animated ‘explosion’ was presented at the end of each trial with its final radius indicating reach accuracy (not shown). Also, during *training* sessions a 4-bar histogram summary, shown after each block (84 trials), informed the participant about his or her ability to initiate movements, move, aim and reach the target (adopted from [16]). **c.** The reaching workspace used for mapping performance. The locations of the 8 targets are indicated by small open circles and are specified by angular coordinates relative to the centre. An example of the hand located at the 90^o^ target is shown. Participants made 5 cm reaches to each target from 8 start locations (indicated, for the example target, by small black dots and arrows), which were also specified in angular coordinates relative to the particular target. Note that the start coordinates therefore correspond to intended movement direction. The dashed circle indicates the extent of the mapped workspace, centred 24 cm in front of the headrest

**Fig. 3:**
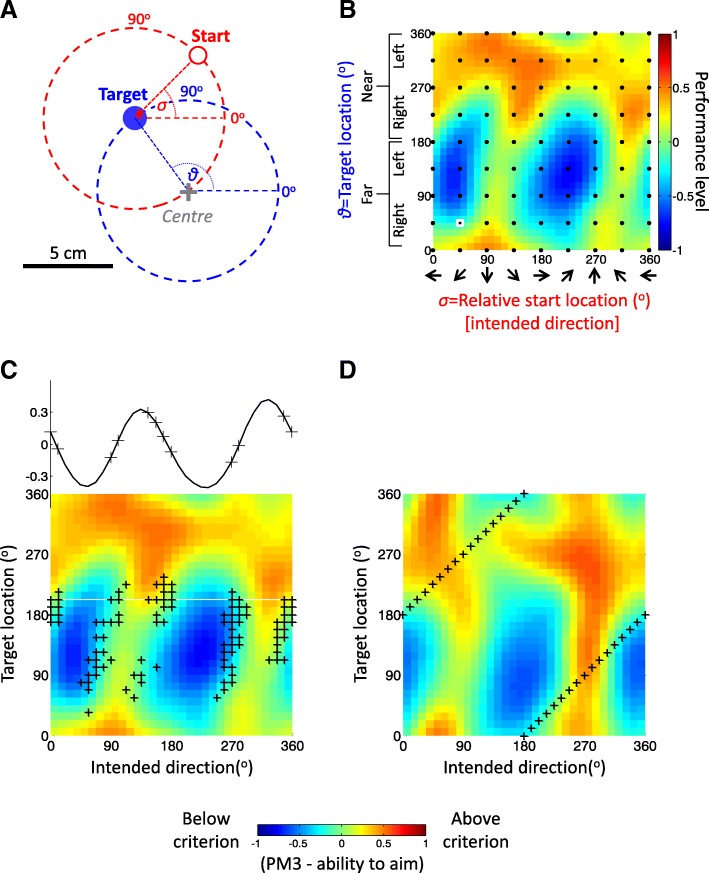
Performance mapping and selection of movements. **a.** The workspace for mapping motor performance is defined by all possible targets angles ϑ (targets 5 cm away from the workspace centre; blue dashed line) and all the possible start angles σ from each target (red dashed line, indicating the possible start locations relative to the target shown as a blue disc). Note that start locations define movement directions. **b.** An example of a 32 × 32 ϑ-by-σ performance map (interpolated and smoothed from the 8 × 8 performance metric PM3, indicated as black dots). Reddish colours indicate good performance, bluish colours poor performance. Note that opposite edges of the map are in fact contiguous, due to the circularity of angular data (0^o^  = 360^o^). The small white square, bottom left of map, indicates the coordinates of the start-to-target movement example shown in panel **a** (ϑ = 135, σ = 45; red Arrow). **c.** Example of performance-based selection of practiced movements (‘+’) according to the steepest gradients principle. The example selection shown was based on the PM3 map from a representative patient (also shown in panel **b**). Selected movements are located at regions where performance changes rapidly from higher to lower levels (relative to the participant’s overall performance). The graph across the top depicts the performance gradient measured at target direction of 202.5^o^; as indicated by the white line on the map) and the corresponding selected movements (+). Note that selection is based on the vector sum of the local gradients across both map dimensions. Note also that the training sets for the study consisted of weighted selections from the PM2 and PM3 maps (see text and [16] for more details). **d.** The coordinates of centre-out movements which were selected for the control group (‘+’)

For both groups, the study period was divided into 3 phases (Fig. 2a). The initial baseline phase consisted of five sessions lasting 1–1.5 h: two identical CAs, a parameter *tuning* session, a performance *mapping* assessment and an MRI brain scan (for MR-eligible individuals). The main training phase comprised 4 sessions per week for 5 consecutive weeks. In each week, 3 *training* sessions were followed by a *mapping* session. Data from the final *mapping* session of the training phase, and from a following CA (conducted within 2–4 days post-training), served to evaluate post-training outcomes. A final CA and *mapping* session were run 4 weeks later (follow-up phase). The two groups differed only in the selection of movement conditions during *training* sessions; all other session types were identical in both groups (see Fig. 3 and below).

### Clinical assessments (CA)

The therapist who conducted the CAs and screening was blinded about the participant’s group allocation and ongoing test results. Sensorimotor assessment included the UL FMA-UE [27], the MRC Muscle Power scale (MRC-MPS) [28], the Modified Ashworth Scale (MAS; elbow flexors) [29] and the new Motor Assessment Scale – UL items (nMAS-UE, items 6–8) [30]. Activities of daily living were assessed using the Barthel index (BI) [31]. The primary outcome measure was the FMA-UE score, as it has been reported to be sensitive to robot-assisted UL therapy (e.g. [16]). The other clinical assessments served as secondary clinical outcome measures.

Baseline CA scores were computed as the mean clinical scores across the two baseline tests.

### Robot-assisted sessions

For both groups, during all the robot-assisted task sessions (*tuning, mapping* and *training* sessions) the participants sat in front of a horizontal display holding the handle of a robotic manipulandum (vBot [32]; Fig. 2b) and attempted to perform 5 cm point-to-point reaching movements within an individually-set allotted time. Assistive and guiding forces were provided as needed by the vBot throughout the movement, using an algorithm adapted from [16] and described elsewhere [22]. Briefly, the assisting force (*Assist*) was provided in the direction towards the target and depended on the momentary speed towards the target relative to the speed expected in a minimum jerk trajectory [33], at the particular time point given the allotted movement time (which was set individually). The guiding force (*Guide*) impedes deviation from moving towards the target at each moment and depended on the amount of momentary deviation. Each session started with 24 initial practice trials.

During *tuning,* the allotted movement time and stiffness of the vBot’s guiding force were individually adjusted based on a revised version of [16] and the final adjusted values were then fixed for each participant throughout the rest of the robot-assisted sessions [22].

In *mapping* (and *tuning*) sessions, performance was assessed across 64 planar reaching movements, defined by all combinations of 8 targets, located equidistant 5 cm away from the workspace’s centre, and 8 target-related start locations, equidistant 5 cm around each target (Fig. 2c). In each *mapping* session, these 8-by-8 conditions were repeated 5 times in pseudo-random order.

Details of the procedure of mapping performance across movements can be found elsewhere [22]. Briefly, the mapping was based on performance measures (PM) adopted from [16] of ability to move (PM2) and to aim towards the target (PM3) relative to individually-set performance criterion. Negative PM values indicate impaired performance and positive values indicates excelling the individually-set expected performance. Specifically, PM2 is the sum of a movement ability parameter *pm2*, computed across the individually-allotted movement time. If at time point *i*, movement towards the target is slower than expected, or is abnormally fast, *pm2*(*i*) is negative and is equal to the robot power applied in the direction of the target:

$pm2(i)={F}_y(i)\dot{y}(i)$, in the direction towards the target, axis *y*, where F_y_ is the *Assist* force, and $\dot{y}$* is velocity*.

Conversely, at time points when the movement is at - or faster than - the expected speed *pm2*(*i*) is positive and reflects the difference between the expected and actual movement displacement along the trajector*y*:

*pm*2(*i*) = *y*(*i*) − *y*_*m*. *j*_(*i*); where *y*_*m*. *j*_(*i*) is the expected progress towards the target at time point *i*, given an allotted movement time and assuming a minimum jerk trajectory [33].

PM3 is proportional to the difference between root-mean-square (rms) deviation and the tolerated rms deviation, *x*_*rms*_, (individually set), across all movement time points *i*, and normal to direction towards the target (axis *x*):

$$ PM3\propto {x}_{rms}-\sqrt{\frac{1}{N}{\sum}_{i=1}^Nx{(i)}^2} $$

PM2 and PM3 were separately mapped in an angular coordinate framework, specifying target location and relative start location (namely, intended direction; Fig. 3a). That 8 × 8 grid was then interpolated (using a Gaussian-process regression [34]) to create a 32 × 32 map (Fig. 3b). For the test group, the performance maps from each *mapping* session were the basis for selection of training movements for the following three *training* sessions (Fig. 2a and see below).

Each *training* session involved 504 reaches. The control group was trained with 5 cm centre-out reaches to one of 32 equidistant targets, arrayed around the central start position (pseudo-randomised; these define a consistent set of movements, regardless of the performance map; Fig. 3d). For the test group, the training movements were also 5 cm planar reaches within a wide workspace, defined by pairs of target location (any location lying on an invisible 5 cm radius circle centered 24 cm from the body, in the midline i.e. on the same circle as the centre-out targets) and start location (to specify a specific reach direction towards that particular target; Fig. 3a), and were selected according to the “steepest gradients” principle (Fig. 3c), based on the participant’s performance map created in the most recent updated *mapping* session. For each of the current PM2 and PM3 maps, 2D gradients were computed across the smoothed 32 × 32 map and a subset of 102 movements corresponding to the coordinates of the top 10% of gradient values was chosen. The training movement for each trial was then pseudo-randomly selected from one of these two movement subsets. The two PM maps captured different aspects of impairment (i.e. the ability to move in good speed and the ability to aim to the target) which could differ in severity. Therefore, to allow the training selection to be biased to the metric reflecting more severe impairment, the fraction of movements selected from each of the two subsets was based on the ratio of the mean PM scores computed across the worst 25% in each map. This process of movement selection was repeated every 21 trials after updating the PM map data with the latest performances scores; a new performance map and set of movements selection was created in a new *mapping* session at the end of each week.

Three measures of performance at the robot-assisted task (collected during the *mapping* sessions) served as secondary task-related outcome measures additional to the above-mentioned secondary clinical outcome measures. These included the overall level of the forces applied by the robot (*Assist* and *Guide*; [22]; averaged across 5 repetitions of each of the 64 assessed movements) and the mean movement end errors (the average distance of the final hand position from the target). Note that values of these measures can only indicate level of impaired performance (as the values converge to zero force (or error) for well-executed movements). Note also that the end error measure is compromised by the robot assistance and by the time limit on trial duration. We chose not to include PM2 and PM3 as secondary outcome measures because they are not reliably comparable across subjects, as they depict performance relative to individualised criteria and they are not bounded by minimal or maximal levels and hence cannot be normalised. They are also confounded by the robot assistance and guidance forces.

### Analysis

Statistical analysis was conducted using SPSS [35]. Normally distributed data, confirmed via Shapiro-Wilk test for normality (*p* ≥ 0.05), were compared using parametric tests (2-tailed; Student’s t-test (test group: df = 6; control group: df = 8) or repeated measures ANOVA). Otherwise, non-parametric tests were used (2-tailed; Wilcoxon signed rank test for within-group comparisons and Mann-Whitney U Test (exact) for between-group comparisons). Homogeneity of variance was decided based on Levene’s Test (*p* ≥ 0.05).

For one measure, namely the post-training FMA-UE score, the training effect (score change) correlated significantly with baseline data. In that case, the data of the two groups were compared using one-way analysis of covariance (ANCOVA), after confirming that all the assumptions were validated, including homogeneity of error variances confirmed by Levene’s Test and normally distributed residuals based on Shapiro-Wilk test and Q-Q plots.

Since there was a large variation across participants in task performance and required levels of robot assistance, the task-related training effects were also analysed in terms of percent of change relative to baseline. This approach could not be applied for the analysis of the effect of training clinical assessments since the clinical scales are ordinal.

The overall task difficulty (in the *mapping* sessions) was evaluated by the mean values of the secondary task-related outcome measures (*Assist*, *Guide* and end error), across all the 64 assessed movements, while the variation of task difficulty across movements was evaluated by the standard deviation across these movements.

A *mapping*-vs-*training* difficulty index -the relative difference in difficulty between the *mapping* sessions and their adjacent *training* sessions (see Results) - was evaluated as the mean difference in the force (*Guide* or *Assist*) provided during these sessions:

1 $$ \Delta dif=\frac{1}{2n+1}\left[{\sum}_{w=1}^5\frac{\left({F}_{training}\left(s(w)-1\right)-{F}_{mapping}\left(s(w)\right)\right)}{F_{mapping}\left(s(w)\right)}+{\sum}_{w=1}^4\frac{\left({F}_{training}\left(s(w)+1\right)-{F}_{mapping}\left(s(w)\right)\right)}{F_{mapping}\left(s(w)\right)}\right], $$

where *F*_*mapping*_ and *F*_*training*_ are the applied *Guide* or *Assist* forces during the *mapping* and *training *sessions, respectively; *s(w)* – the number of *mapping* session *s* at week *w* within the training phase.

## Results

### Baseline

The severity of UL impairment at entry to the protocol, and the locations and extent of the participants’ brain lesions are summarised in Table 1. For participants C5, C6 and T3, limited anatomical details were available; for the remainder, the lesion location was based on a current T1-weighted structural MR image.

**Fig. 4:**
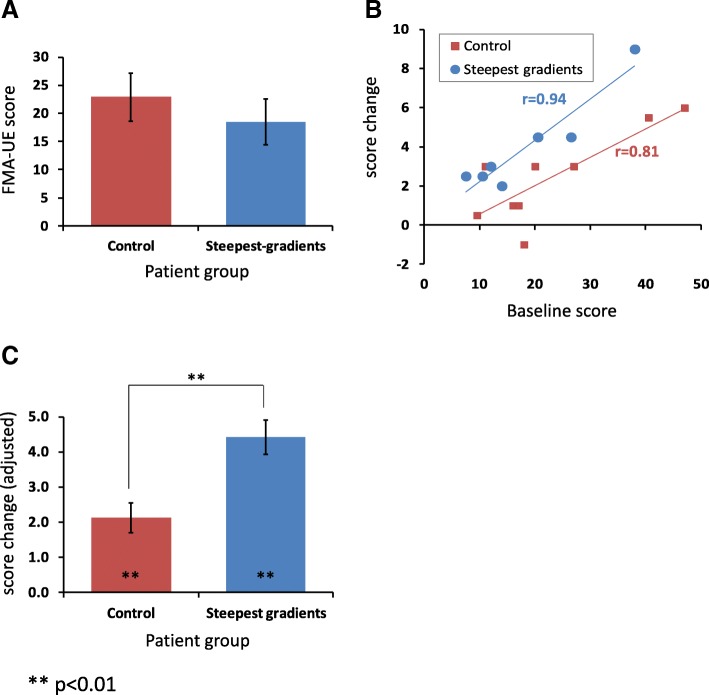
FMA-UE scores. **a** Baseline scores of the two groups did not significantly differ. **b** Correlation between baseline score and training-induced change. **c** Post-training score change relative to baseline (plotting the adjusted estimated marginal means by controlling for baseline scores as a covariate). Error bars in panels **a** and **c** indicate standard error of the mean

The FMA-UE baseline scores of the two groups were comparable (Fig. 4a; *p* = 0.475), as expected from the stratified group allocation. Likewise, the two groups did not significantly differ in baseline values of any of the secondary outcome measures (both clinical and task-related (see Additional file 1: Tables S1 and S2). There was a floor effect for the nMAS-UE scale where most participants (6 control and 5 test group) had minimal scores (≤1.5 out of 18).

### Task performance variability

Generally, in all the *mapping* sessions, the raw PM data varied systematically across movement conditions. Linear correlation between the raw mapping data and their Gaussian Process interpolation regression fit was generally high for each map. The median variance explained (r-squared values) exceeded 0.6 and the lower quartile was higher than 0.43 across groups and PM maps (i.e. 75% of the cases had r-squared > 0.43). Low r-square (< 0.2) values were found in some sessions for 3 better-performing patients (FMA-UE ≥ 38) and once each for two more severely impaired patients. In each of these cases the PM maps were ‘flattened’, with good or bad performance respectively.

### Post-training effects

Following training, all but one participant (participant C9) improved in their FMA-UE scores showing an average increase of 3.13 ± 0.6 relative to baseline. A 2 by 2 repeated measures ANOVA, with between-subject factor group (test, control) and within subject factor session (baseline, post-training) confirmed a significant main effect of session (F (1,14) = 29.456, *p* < 0.001, partial *η*^2^ = 0.68.

On average, the test group’s improvement exceeded that of the control group (with increases in FMA-UE of 4.00 ± 0.9 and 2.4 ± 0.8, respectively), although the ANOVA failed to show significant interaction between the session and group effects (F (1,14), *p* = 0.21, partial *η*^2^ = 0.11). Still, further inspection of the data revealed a strong linear correlation between improvement (baseline-to-post training score change) and the baseline level -for both test and control groups (Fig. 4b; Pearson’s rho = 0.94 and 0.81, respectively; *p* < 0.01 in both cases). Hence, to compare the post-training effects while controlling for this confounding effect of the baseline scores, a one-way ANCOVA was conducted with group (test, control) as an independent variable, post-training change in FMA-UE score from baseline as the dependent variable, and baseline FMA-UE score as the covariate. This analysis revealed a highly significant group effect (F (1,13) = 12.717, *p* = 0.003) and a large statistical effect size(partial *η*^2^ = 0.5). The ANCOVA-based estimated marginal means of the score change confirm the superior improvement for the test group compared to control (4.42 vs. 2.12, respectively; Fig. 4c).

For both groups, none of the secondary clinical outcome measures showed significant change from baseline (see Additional file 1: Table S3).

Intriguingly, the task-related performance measures (*Assist, Guide* and end-error) taken in the post-training *mapping* session did not mirror the group effect of training on the clinical FMA-UE scores. For each performance measure we conducted a 2-by-2 repeated measures ANOVA with group (test, control) as a between-subject factor and session (baseline, post-training) as a within-subject factor), comparing the mean performance values across the 8 × 8 movements tested conditions (see Additional file 1: Table S4). Although both groups showed a trend towards a within-subject improvement with the task (i.e. reduction in lateral guidance and forward assistance from the robot, and in the end errors; shown as negative change values in Table 2) a significant main effect of session was found only for the end-error measure (F (1,14) = 15.4, *p* = 0.002, partial *η*^2^ = 0.52; *Assist*: *p* = 0.43; *Guide*: *p* = 0.069). Moreover, even for the end-error measure there was no significant interaction between the group and session effects (F (1,14) = 0.16, *p* = 0.70, partial *η*^2^ = 0.01), indicating comparable improvement in both groups (*Assist* and *Guide*: *p* > 026). Similarly, the variation of task performance (computed as standard deviation across the 64 movement conditions) showed a trend of reduced variation after training (negative change values in Table 2), which was marginally significant only in the case of the end-error measures (F (1,14) = 4.66, *p* = 0.049, partial *η*^2^ = 0.25). There was no interaction effect between the session and group factors (*p* > 0.065 (see Additional file 1: Table S5)).

**Table 2:** Post-training change in task performance relative to the baseline

		% Change^a^		
		*Guide*	*Assist*	End-error
mean^b^	Test	−14.5 ± 20.2	−13.5 ± 34.4	−16.9 ± 13.3
	control	−21.6 ± 21.6	−24.4 ± 37.7	−19.3 ± 19.9
	p (between groups)	0.51^d^	0.56 ^d^	0.79 ^d^
variation^c^	test	−7.5 ± 45.8	− 4.5 ± 41.2	− 10.4 ± 26.9
	control	−23.6 ± 28.6	−25.2 ± 35.1	−19.6 ± 23.0
	p (between groups)	0.92 ^e^	0.30 ^d^	0.47 ^d^

^a^Mean ± SD change relative to baseline (across subjects). Negative change indicates improvement

^b^Mean across the 8 × 8 movement testing conditions

^c^The standard deviation across the 8 × 8 movement conditions

^d^Independent samples T-test, 2-tailed; equal variance assumed

^e^Mann-Whitney U Test

Unlike the FMA_UE data, there was no consistent correlation between the post-training change in performance and the baseline performance values for any of the task performance measures. However, in 4 of the 6 task performance measures there was a high correlation (Pearson’s rho> 0.7) between the baseline score and the post-training change (but inconsistently across groups) or the absolute value of the change. Therefore, to control for possible between-subject masking of within-subject training effects we also compared the percent of within-subject change in task performance relative to the baseline level. However, as shown in Table 2, the % change of performance (or its variation) after training did not differ significantly between the two group for any of the task-performance measures.

In summary, despite the highly significant differential improvement in impairment scores (FMA-UE), the two groups did not differ significantly in their improvement in the task itself. This unexpected finding might reflect a methodological confound in how we assessed task performance. The potential confound lies in the fact that the *training* and *mapping* sessions involve different sets of movements. For the control group, *training* is restricted to 32 movements, all starting at the workspace centre. During *mapping* sessions their performance is assessed across 64 movements, only 8 which start at the centre, and some of these may be more demanding for any one participant than the centre-out reaches, especially movements requiring larger elbow extension. In contrast, for the test group, the *training* movements are selected to be challenging (the performance-based steepest gradients principle), and vary in both start and target locations, whereas the *mapping* performance involves the same 64 movements as for the control group, some which are likely to be easier than the training set.

**Fig. 5:**
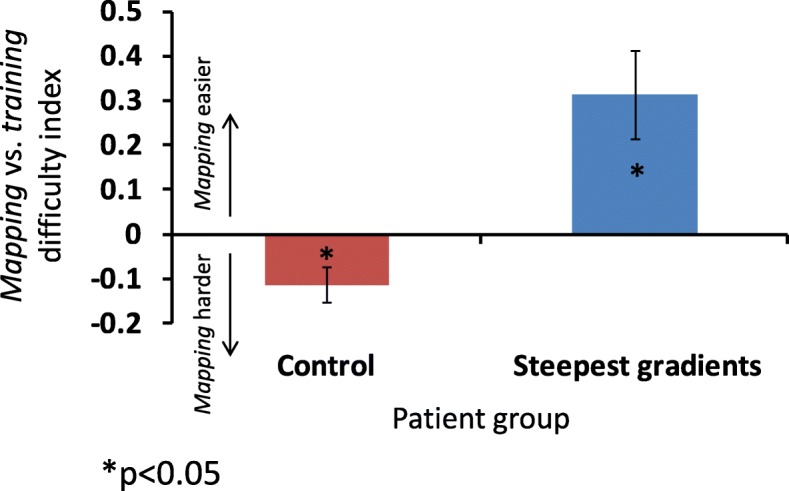
The difference in overall difficulty between the *mapping* sessions and the *training* sessions for the two groups, assessed with mean *Guide* force levels. Error bars indicate standard error of the mean

Hence the two groups may show opposite differences between *mapping* and *training* sessions in the overall level of task difficulty, which might lead to differences in strategy and motivation, potentially masking genuine learning effects. To evaluate this possibility we looked at the *mapping* vs. *training* difficulty, namely, the difference in the mean *Assist* and *Guide* levels between each *mapping* session and its adjacent *training* sessions (see Eq. 1, Methods). Indeed, the *mapping*-vs-*training* difficulty index for the guiding force differed from zero for both groups (Fig. 5; test group: *p* = 0.018 (one-sample Wilcoxon signed rank test); control group: *p* = 0.025 (t (8) = − 2.75, one-sample t-test)), indicating that the guiding force levels differed between the two session types. Importantly, as predicted, the differences were opposed between the two groups. For the test group, the *training* sessions required overall more guiding force than in the *mapping* sessions, and vice versa for the control group. A trend with the same effect was seen for the *Assist* measures, but this was not significant (test group: t (6) = 1.42, *p* = 0.207; control group: t (8) = 1.01,*p* = 0.343).

### Follow-up

Four weeks after the end of training the majority of the participants still maintained above-baseline FMA-UE scores (6 out of the 7 test participants, at least 2 points above baseline; 7 out of the 9 participants: at least 1 point above baseline). A by 2-by-2 repeated measure ANOVA, with session (baseline, follow-up) as a within-subject factor and group as a between-subject factor, confirmed a main effect of session (F (1,14) = 16.37, *p* = 0.001, partial *η*^2^ = 0.54). However, there was no significant interaction between the session and group effects (F (1,14) = 0.04, *p* = 0.843, partial η^2^ = 0.003), implying that the score change at the follow-up session was comparable in both groups (test group: 2.71 ± 1.15; control: 3 ± 0.87 (mean and s.e.m.)). Note that - unlike for the post-training data - here the amount of score change from baseline did not significantly correlate with the baseline scores (*p* > 0.17). Further inspection reveals that this is mainly due to increase in within-group variability in the follow-up session, especially in the test group where three participants clearly continued to improve whereas the scores of two others sharply declined between the post-training session and the follow-up (see Additional file 2: Figure S1).

Similar 2-by-2 ANOVAs of the follow-up data of each of the task-related measures replicate the post-training results, with largely non-significant training effects except for a main session effect on end-error (see Additional file 1: Table S6). The post-training improvement from baseline was maintained only for the mean end-errors measure (main effect of session: F (1,14) = 7.78, *p* = 0.014, partial *η*^2^ = 0.36), and even in that case, there was not significant interaction between the group and session effects (F (1,14) = 3.78, *p* = 0.072, partial *η*^2^ = 0.21). The trend of post-training within-subject improvement in end-in error variation was not maintained here (*p* = 0.21).

## Discussion

We present a novel method of robot-assisted training for upper-limb motor impairment that is based on individualized selection of movements. Specifically, training with movements located within regions of steep transition from high to low performance on the individual’s performance maps led to noticable improvement in FMA-UE clinical scores compared to training with standard (non-individualised) centre-out movements. The statistical effect size was large (partial *η*^*2*^ = 0.5), and highly significant (*p* < 0.003). To our knowledge this is the first demonstration of boosting UL motor capacity by the individualised selection of robot-assisted training sets.

The large statistical effect size is particularly striking, especially given that it was demonstrated on a population of moderate to severe chronic hemiparetic participants, who tend to show poorer recovery [36, 37]. Importantly the benefit was demonstrated in terms of clinical assessment (FMA-UE scores) suggesting that our method is effective in reducing UL motor impairment, rather than being limited to task-related training effects. Although there was no improvement in the secondary clinical outcome measures, potentially due to the rather crude scale for MAS and BI and a floor effect for UL nMAS (see Additional file 1: Table S3), the large statistical effect size for the primary clinical outcome measure (FM-UE) suggests it is a step in the right direction. Likewise, given the small sample size, the possibility that the steepest gradient effect is overestimated cannot be ruled out, but the large statistical effect size justifies further trials with large sample sizes to confirm the therapeutic effect.

Overall, the positive effect of both performance-based (steepest gradients) and standard robot-assisted training on improving clinical scores was maintained for at least 1 month for most participants, echoing previous studies [38]. However, in our study the superior benefit of steepest gradients-based training over and above the standard was not retained for about half the group. Understanding this null effect requires further study. One potential explanation is that steepest-gradients-based training may boost the rate of improvement, perhaps due to the focus on movements that optimise improvement, while minimizing time spent on movements that do not contribute to improvement, but this higher rate of gain may be accompanied with less efficient consolidation. It is possible, therefore, that longer training with more repetitions of steepest-gradients -selected movements would increase the early gain seen in steepest-gradients-based training, and also improve its long-term consolidation. Encouraging support for this prediction is our finding of a tight positive relationship between the baseline level and improvement in FMA-UE score. This suggests that longer training would lead to some acceleration of improvement, as individual trainees move from their baseline to higher and higher scores and derive greater benefit from additional training. Combining steepest-gradients-based training and transcranial direct current stimulation (tDCS) over the ipsilesional motor cortex may also promote consolidation and further enhance the effect, based on the effectiveness of that method in enhancing motor learning consolidation in healthy participants [39] and its applicability for use in stroke patient population [40]; however, clinical evidence is still weak [41].

Considering the severity of hemiparesis for some of the participants, the 5-week training period of our proof-of-concept study may have been insufficient. This might account for the fact that despite strong evidence for superior benefit of the steepest gradients training approach, the overall post-training change in FMA-UE score did not reach the criterion for a minimum detectable score change (5.2 points) [42]. However, the 3 least impaired individuals did exceed this threshold. Note also that -based on the linear relationship between post-training improvement and baseline scores - surpassing this “minimum detectable change” after 5 weeks of steepest-gradient training would be predicted for individuals with baseline FMA-UE scores of at least 24 points, whereas a baseline level of at least 31 points would be needed to benefit from control training. In other words, we expect our protocol to have benefit for a wider range of abilities. Of course, larger sample sizes are needed to verify this prediction.

Another issue is the unexpected similarity of the groups in their post-training improvement in the task, despite clear evidence for an enhanced benefit for the test group in clinical scores (FMA-UE). Comparing the mean guiding force provided in the *mapping* and *training* sessions revealed that the relative difficulty of the *training* vs. *mapping* sessions was opposite in the two groups (recall that the *mapping* conditions were identical between groups). Specifically, the test group required significantly more guidance in the *training* sessions than in *mapping* sessions, and vice versa for the control group. Hence we suggest that the test group tended to compromise their performance during the easier *mapping* sessions, as even with less effort they could perform as well as–or even better–than in the *training* sessions, whereas control participants attempted to enhance their effort during the *mapping* sessions, in order to maintain their overall performance level similar to that in the more difficult *training* sessions. Together these opposite effects might cancel out any measured improvement of the test group’s task performance that was expected from their improved clinical scores. This indicates a methodological limitation of this study and an important lesson to be considered when designing future studies on task-related learning (see also [43]). One way to minimise such performance biases in future studies would be to use blocked (rather than randomised) trials of different movements during the *mapping* sessions. This would encourage strategies to maximise performance for each assessed movement, allowing the best performance for each action to be mapped. Nevertheless, considering that the objective of our study was to improve the clinical outcome of UL robot-assisted therapy, task-related effects are only of secondary interest.

However, the finding that the steepest-gradients-based training was more difficult on average also raises the possibility that the key point for superior training outcome is the overall level of difficulty of the training rather than the specific set of trained movement. Clarifying this requires further research with multiple training conditions where the level of difficulty and the selection of training movements are manipulated. A similar issue regards to the choice of the specific conditions of the control intervention. Centre-out training was chosen because it is commonly used in robot-assisted intervention for planar UL movements [7, 16, 26] and it involves a range of movements. However, in our study the movement displacement (5 cm) was shorter than in previous studies (usually ≥10 cm). The 5 cm distance was chosen to enable equal movement displacement in the two groups: since the steepest gradients method requires full mapping of performance across a wide range of start positions, this imposes a limitation on the maximum movement displacement (around 5–8 cm; constrained by the comfortable reach distance of a typical adult participant). Potentially the effect of training with such a small movement displacement might be sub-optimal, but this possibility is not supported by evidence from large sample-size trials that provided intensive training with larger UL movements in chronic stroke hemiparetics. For example, 6 weeks of intensive training with larger planar movements led to improvement of between 1 and 3 points in FMA-UE score (Fig. 2 panels A & B in ref. [7]). Similarly, 4 weeks of intensive training with large 3D movements led to a 2.6 score change [44]. These results are comparable with the overall improvement found for the control group in our study (2.4 score change). Still, further study with larger sample size will be needed to exclude the possibility that our finding of superior effectiveness of the steepest gradients training is related to the particular selection of the centre-out control condition.

Finally, updating the performance maps required a full weekly session. It would have been desirable to update the maps during the training, to save time. However, although technically possible, the steepest gradients training is always limited to specific sub-regions of the map and so using only the training movements to update the performance map is likely to lead to some regions being undersampled, and this may distort the estimate of the steepest gradient profile. Hence, to avoid biases we chose to include a periodic assessment of performance across the full workspace.

## Conclusions

This study provides a proof of concept for the principle of using steepest performance gradients in selecting robot-assisted training of the upper limb after stroke. The large statistical effect size encourages further clinical trial work with large sample sizes. Further work may also help to optimise the method. The next step would be to evaluate whether longer periods of individualized training can extend its benefit for longer term retention and also increase training effectiveness. It would also be valuable to extend the range of movement (e.g. in other planes). Finally, the principle of steepest performance gradients is a general principle for selection of training based on a detailed performance profile. It is left for future studies to evaluate whether this principle would be advantageous for other motor impairments (e.g. gait, or hand movements), other devices (e.g. exoskeletons), or even for training and assessment without assistive force devices (e.g. with performance based on motion tracking).

## Additional files

Additional file 1: This file contains two tables summarising the secondary outcome measure results. Tables S1 and S2 summarise the baseline levels of all the clinical task-related performance measures, respectively. Table S2 and Table S3 summarises the post-training change of the clinical measures (PDF 191 kb). Tables S4 and S5 summarise the post-training effects on the mean and variation of the task performance, respectively (in terms of repeated measures ANOVA). Table S6 summarises the task-related effects during the follow-up session (in terms of repeated measures ANOVA). (PDF 190 kb)

Additional file 2: Figure S1. This file contains graphs showing the post-training and follow-up change in individual FMA_UE scores. (PDF 83 kb)

## Abbreviations

BI: Barthel index
CA: Clinical assessment
FMA-UE: Upper extremity part of the Fugl-Meyer assessment
MAS: Modified Ashworth Scale (elbow flexors)
MRC-MPS: MRC Muscle Power scale
nMAS–UE: New Motor Assessment Scale –upper extremity items
PM2 & PM3: Performance measure parameters, adopted from (16), measuring the ability to move and aim, respectively
UL: Upper limb
vBot: Robotic manipulandum
