# Correction to: Boosting robot-assisted rehabilitation of stroke hemiparesis by individualized selection of upper limb movements – a pilot study

**DOI:** 10.1186/s12984-019-0521-0

**Published:** 2019-04-15

**Authors:** Orna Rosenthal, Alan M. Wing, Jeremy L. Wyatt, David Punt, Briony Brownless, Chit Ko-Ko, R. Christopher Miall

**Affiliations:** 1 School of Psychology, University of Birmingham, Birmingham, B15 2TT UK; 2 School of Computer Science, University of Birmingham, Birmingham, B15 2TT UK; 3 School of Sport, Exercise and Rehabilitation Sciences, University of Birmingham, Birmingham, B15 2TT UK; 4 West Midlands Rehabilitation Centre, Birmingham, B29 6JA UK

**Correction: J Neuroeng Rehabil**

**https://doi.org/10.1186/s12984-019-0513-0**

The original article [1] contained a minor error in the following sentence in the Discussion:

*‘… a baseline level of at least 31 points would be needed to benefit from control training.’*

The aforementioned sentence has now been corrected to state the following:

*‘… a baseline level of at least 42 points would be needed to benefit from control training.’*
