# Supplementary material for: Boosting robot-assisted rehabilitation of stroke hemiparesis by individualized selection of upper limb movements – a pilot study
Source: J Neuroeng Rehabil. 2019 Mar 20;16:42. doi: 10.1186/s12984-019-0513-0 (PMC6425657; doi:10.1186/s12984-019-0513-0)
Supplement: Supplementary file 1 — This file contains two tables summarising the secondary outcome measure results. Tables S1 and S2 summarise the baseline levels of all the clinical task-related performance measures, respectively. Table S2 and Table S3 summarises the post-training change of the clinical measures (PDF 191 kb). Tables S4 and S5 summarise the post-training effects on the mean and variation of the task performance, respectively (in terms of repeated measures ANOVA). Table S6 summarises the task-related effects during the follow-up session (in terms of repeated measures ANOVA). (PDF 190 kb) [file 12984_2019_513_MOESM1_ESM.pdf]

SUPPLEMENTARY TABLE S1:

Secondary clinical outcome measures – baseline values[a]

Group	MRC-MPS	MAS (elbow flexors)	nMAS - UE	BI
**Control**	49.7 (5.8)	1.22 (0.62)	2.67 (4.02)	18.2 (1.8)
**Test**	39.9 (14.9)	1.28 (0.49)	1.93 (2.96)	16.8 (3.2)
**p** (between groups)	0.14[b]	0.91[c]	0.62[c]	0.30[b]

[a]Group means (and standard deviations)

MRC-MPS: MRC Muscle Power scale (score range: 0-70); MAS: Modified Ashworth Scale (0-4); nMAS-UE: Upper extremity part of new Motor Assessment Scale (0-18), BI: Barthel index (0-20). For all scales—except MAS – higher score indicates lower severity.

[b]Independent samples T-test 2-tailed (Test group: df=6; control group: df= 8)

[c]Mann-Whitney U Test, 2-tailed, exact

SUPPLEMENTARY TABLE S2:

Secondary task-related outcome measures – baseline values[a]

Group	Mean[b]			Variation[c]		
	Assist(N)	***Guide***(N)	**end error** (cm)	***Assist***(N)	***Guide***(N)	**end error** (cm)
**Control**	2.02 (1.21)	1.81 (1.14)	0.82 (0.28)	1.71 (1.17)	1.23 (0.89)	0.43 (0.20)
**Test**	2.18 (1.65)	2.02 (1.64)	0.89 (0.39)	1.49 (1.12)	1.29 (1.09)	0.44 (0.25)
**p[d]** (between groups)	0.82	0.77	0.72	0.69	0.90	0.93

[a]Group means (and standard deviations)

[b] Mean performance across the 8x8 movement testing conditions

[c] The standard deviation of performance computed across the 8x8 movement conditions.

[d] Independent samples T-test 2-tailed (Test group: df=6; control group: df= 8)

SUPPLEMENTARY TABLE S3:

Secondary clinical outcome measures: post-training change from baseline[a]

Group	MRC-MPS	MAS (elbow flexors)	UL nMAS	BI
**Control**	-0.4 (6.7) p=0.71[b]	-0.1 (0.5) p=0.56[c]	1.9 (3.8) p=0.41[b]	0.19 (0.5)[d] p=0.50[b]
**Test**	2.6 (4.3) p=0.17[c]	0.07 (0.19) p=0.25[b]	1.8 (2.7) p=1.0[c]	-0.5 (1.1) p=0.28[c]

[a] Group means (and standard deviations)

[b] Related-samples Wilcoxon Signed Rank Test (exact sig., 2-tailed)

[c] Paired T-test (Test group: df=6; control group: df= 8 --except for BI where df=7)

[d]Missing data from one aphasic participant (due to unavailability of interpreter for his speech)

SUPPLEMENTARY TABLE S4

Task-related training effects on post-training mean performance[a]: 2x2 repeated measure ANOVA[b]

	Guide	*Assist*	**End-error**
**Session effect** (within subject)	F(1,14)=3.89, p=0.069 partial η^2=0.22	F(1,14)=0.67 , p=0.43 , partial η^2=0.05	F(1,14)=15.4 , p=**0.002** , partial η^2=0.52
**Group effect**	F(1,14)=0.22 , p=0.65 , partial η^2=0.02	F(1,14)=0.27 , p=0.63 , partial η^2=0.02	F(1,14)=0.08 , p=0.78 , partial η^2=0.01
**Session x group interaction**	F(1,14)=0.76 , p=0.40 , partial η^2=0.05	F(1,14)=1.37 , p=0.26 , partial η^2=0.089	F(1,14)=0.16 , p=0.70 , partial η^2=0.01

[a]Mean performance across the 8x8 movement testing conditions

[b]Repeated measures ANOVA with session (baseline, post-training) as a within-subject factor and group (control, test) as a between-subject factor

SUPPLEMENTARY TABLE S5

Task-related training effects on post-training performance variation[a]: 2x2 repeated measure ANOVAs[b]

	Guide	*Assist*	**End-error**
**Session effect** (within subject)	F(1,14)=0.39 , p=0.54 , partial η^2=0.02	F(1,14)=0.41 , p=0.53 , partial η^2=0.03	F(1,14)=4.66 , p=0.049 , partial η^2=0.25
**Group effect**	F(1,14)=0.25 , p=0.62 , partial η^2=0.02	F(1,14)=0.02 , p=0.89 , partial η^2=0.001	F(1,14)=0.051 , p=0.83 , partial η^2=0.01
**Session x group interaction**	F(1,14)=1.37 , p=0.26 , partial η^2=0.09	F(1,14)=4.01 , p=0.065 , partial η^2=0.22	F(1,14)=0.16 , p=0.70 , partial η^2=0.004

[a]The standard deviation of performance computed across the 8x8 movement conditions.

[b]Repeated measures ANOVAs conducted for each measure with session (baseline, post-training) as a within-subject factor and group (control, test) as a between-subject factor

SUPPLEMENTARY TABLE S6

Task-related training effects in the follow-up session: 2x2 repeated measure ANOVAs[a]

Measure type		*Guide*	*Assist*	End-error
**Mean[b]**	**Session effect** (within subject)	F(1,14)=1.49 , p=0.24 , partial η^2=0.096	F(1,14)=0.038 , p=0.85 , partial η^2=0.003	F(1,14)=7.78 , p=**0.014** , partial η^2=0.36
	**Group effect**	F(1,14)=0.007 , p=0.97 , partial η^2<0.001	F(1,14)=0.003 , p=0.96 , partial η^2<0.001	F(1,14)=0.004 , p=0.95 , partial η^2<0.001
	**Session x group interaction**	F(1,14)=2.51 , p=0.36 , partial η^2=0.152	F(1,14)=0.93 , p=0.35 , partial η^2=0.063	F(1,14)=3.78 , p=0.072 , partial η^2=0.21
**Variation[c]**	**Session effect** (within subject)	F(1,14)= 0.011 , p=0.92 , partial η^2=0.001	F(1,14)=0.016 , p=0.90 , partial η^2=0.001	F(1,14)=1.75 , p=0.208 , partial η^2=0.11
	**Group effect**	F(1,14)=0.003 , p=0.96 , partial η^2<0.001	F(1,14)=0.096 , p=0.76 , partial η^2=0.007	F(1,14)=0.02 , p=0.91 , partial η^2=0.01
	**Session x group interaction**	F(1,14)=0.99 , p=0.34 , partial η^2=0.066	F(1,14)=0.29 , p=0.60 , partial η^2=0.02	F(1,14)=0.96 , p=0.344 , partial η^2=0.06

[a] Repeated measures ANOVAs conducted for each performance measure with session (baseline, follow-up) as a within-subject factor and group (control, test) as a between-subject factor.

[b] Mean performance across the 8x8 movement testing conditions

[c] The standard deviation of performance computed across the 8x8 movement conditions.
