# Supplementary material for: Boosting robot-assisted rehabilitation of stroke hemiparesis by individualized selection of upper limb movements – a pilot study
Source: J Neuroeng Rehabil. 2019 Mar 20;16:42. doi: 10.1186/s12984-019-0513-0 (PMC6425657; doi:10.1186/s12984-019-0513-0)
Supplement: Supplementary file 2 — Figure S1. This file contains graphs showing the post-training and follow-up change in individual FMA_UE scores. (PDF 83 kb) [file 12984_2019_513_MOESM2_ESM.pdf]

SUPPLEMENTARY FIGURE S1:

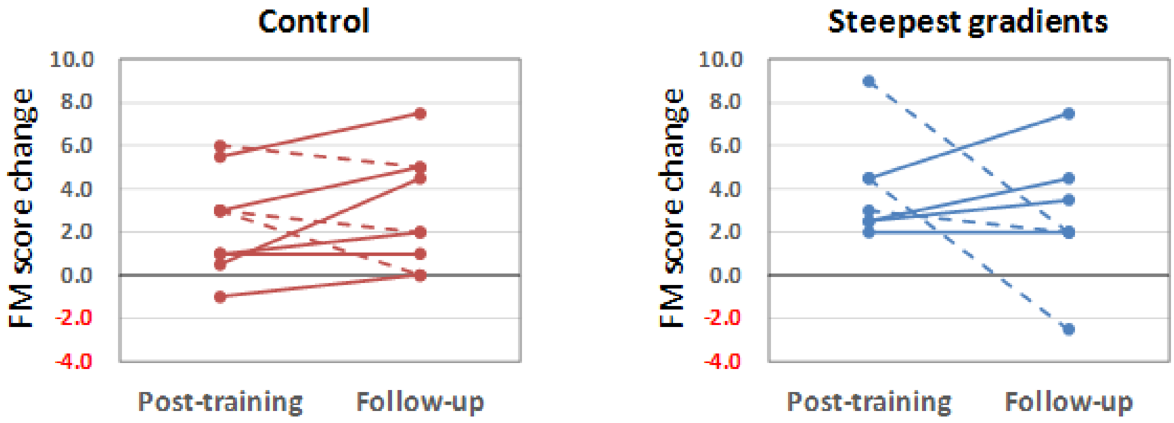

Figure S1. Individual change in FMA-EU scores (from baseline) few days post training and 4 weeks later (follow-up) in the two groups. Dashed lines indicate score decreases in follow-up session compared to post-training.
